# Incidental Abdominal Aortic Aneurysm in the Psoriasis Patient: A Case Report and Review of Literature

**DOI:** 10.22086/gmj.v0i0.1168

**Published:** 2018-12-04

**Authors:** Neda Akhoundi, Taraneh Faghihi Langroud, Kiarash Shafizadeh, Mohamad Javad Jabbarzadeh, Sepehr Talebi

**Affiliations:** ^1^Department of Radiology, Modarres Hospital, College of Medicine, Shahid Beheshti University of Medical Sciences, Tehran, Iran; ^2^Shahid Beheshti University of Medical Sciences, Tehran, Iran

**Keywords:** Aortic Aneurysm, Cardiovascular, Psoriasis

## Abstract

**Background:**

Psoriasis is defined as a chronic inflammatory disease involving keratinocytes hyperproliferation of the epidermis with the acceleration in the epidermal turnover time, which is affected by various factors including genetic, immunologic, and environmental factors. There is a high risk for the development of premature cardiovascular diseases among patients with psoriasis as well as one of the life-threatening events is an aortic aneurysm (AA).

**Case report:**

We report a 60-year-old man with the diagnosed psoriatic disease that an enlargement of the abdominal AA was incidentally found by annual check-up.

**Conclusion:**

Further large-scale studies are needed to find the prevalence of abdominal AA in psoriasis and the appropriate screening time of that in this disease.

## Introduction

Psoriasis is an immune-mediated inflammatory disease that leads hyperproliferation of epiderm, abnormal keratinocytes differentiation, angiogenesis, blood vessel dilation, and excess Th1/Th17, inflammation, and it affects 2-3% of the population worldwide [1]. Because of underlying inflammation of its chronic course, patients are susceptible to some disorders having an inflammatory component, especially cardiovascular and metabolic, collectively christened metabolic syndrome, so psoriasis seems to be a systemic illness rather than mere dermatitis [2]. Patients with cutaneous psoriasis and psoriatic arthritis are more likely developed premature cardiovascular diseases, metabolic syndrome, diabetes type 2, hypertension, and obesity [3]. A systemic review of 90 studies revealed an increased risk for vascular diseases such as peripheral arterial disease, stroke, and ischemic heart disease [4]. Here we report a patient with an abdominal aortic aneurysm (AAA) having no cardiovascular disease in a patient with psoriasis. The inform consnt was taken from the patient before publication.

## Case Presentation

**Figure 1:**
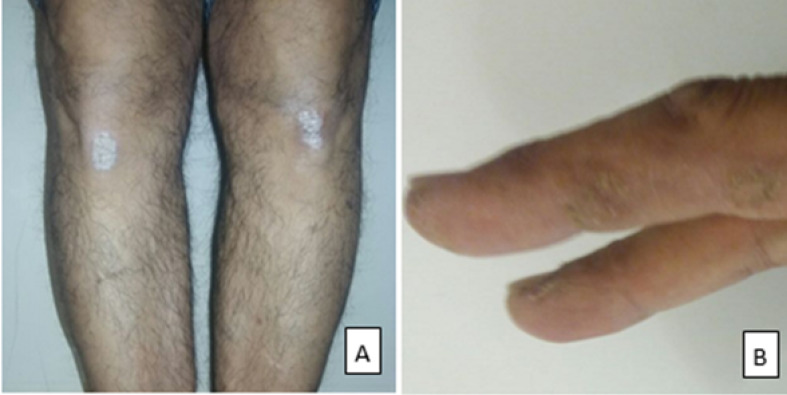
Psoriatic plaques. A: Silvery white scaly plaques on the extensor surface of bilateral knees. B: Scaly plaques were seen on the flexor surface of hand fingers.

**Figure 2:**
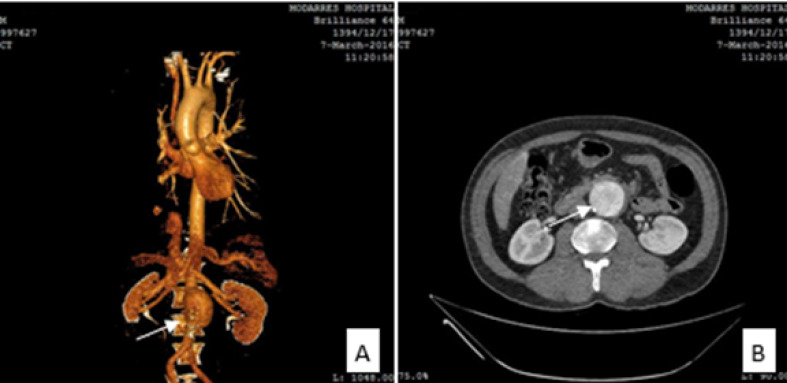
The diagnosed AAA in our patient. A: Chest CT scan with three-dimensional reconstruction indicating AAA. Aortic root, aortic arch, and descending thoracic aorta appear to be normal structure. B: Contrast-enhanced CT scan reveals AAA with evidence of intramural thrombosis.

A 60-year-old man with the diagnosed psoriasis for three years ago that his psoriasis was accompanied with the chief complains of skin rashes in February 2013. Physical exam revealed silvery-white scaly plaques on the extensor surface of bilateral knees, dorsum of the foot, and the flexor surface of hands fingers (Figure-1). He had not any joint involvement. He was treated with topical corticosteroid twice a day and calcipotriol ointment 50 mcg/g once a day but was not in compliance with treatment. Since two months ago he received sulfasalazine 1 gr orally twice, and his plaques seem to be improved with new medication. During yearly check-up, using ultrasonography and computed tomography (CT) angiography of the chest and abdominal aorta, AAA was diagnosed. Aneurysmal dilation of the infrarenal abdominal aorta was 17.3 mm below the origin of renal arteries, and it was seen with length about 59 mm. The bulk of aneurysm formation was more significant on the left side, and the aneurysm formation seems to be eccentric. In the most dilated portion of this mentioned aneurysm, the transverse diameter was 51.4 mm, and its anterior-posterior diameter was 50.3 mm. According to Figure-2, the ascending and descending aorta, as well as aortic root, were healthy. The length of the distal neck from the aorta-iliac bifurcation was approximately measured 21.3 mm. Transverse diameter of the aorta just below the aneurysm was about 14 mm. He did not complain of any symptoms; the electrocardiogram indicated regular sinus rhythm without having conduction disorder, thus to examine other probably cardiovascular diseases, echocardiography was done. Indeed, ejection fraction and function of the aortic valve were in normal range. This case had mild hypertriglyceridemia and hypercholesterolemia that was well controlled by atorvastatin 20 mg daily.

## Discussion

Based on the evidence, psoriasis is considered as an immune system disorder with dermal and circulatory TNF α, INF ɣ, IL 12 elevated levels, and excess T-cell activity [5]. According to one report, eight billion T-cells compared to 20 billion T-cells in the epidermis and dermis of psoriatic plaques was observed in a patient with 20 % body surface area due to psoriasis lesions [6]. On the other hand, AAAs are relatively common and are potentially life-threatening. Pathophysiology of the disease according to surgical specimens of AAA found to be marked the loss of elastin and collagen, thinning of media, chronic adventitial, and medial inflammation with infiltration by lymphocytes and macrophage that may trigger protease activation through cytokines (e.g., TNF α, IL 1, 6, and 8). Based on the evidence, immunoreactive proteins are conspicuously in the abdominal aorta, and this may lead to the increased frequency of aneurysms in this location [7]. Because of inflammatory and immunological causes in both psoriasis and AAA, it may be a causative association between them, but further studies are needed to determine whether psoriasis can cause AAA or not. Heater et al. [8] showed a relationship between S100A8/A9 and psoriasis severity, in vivo vascular inflammation, noncalcified plaque in the coronary arteries, and endothelial cell activation. Regarding these findings, this protein may have a role in linking psoriasis to coronary vascular disease and on the formation of early atherosclerotic plaque that is one of the major causes of AAA [8]. In the study done by Naik et al. [9] with 60 adult psoriasis patients assessing the correlation of inflammation of aorta and psoriasis severity found using FDG PET/CT and activation of neutrophils, they found that the severity of psoriasis was dealing with the inflammation of vessel. Activation and markers of neutrophils increase in psoriasis, and S100A8/A9 protein has a relation with inflammation of vessel and skin disorders [9].

## Conclusion

Our patient was 60-year-old, and his electrocardiogram and echocardiogram showed normal heart structure and function, and AAA was incidentally found on routine screening. Given that one of the life-threatening events is an aortic aneurysm, so it is essential to suspect manage and adequately counsel these patients and let them get away with the belief that their disease is just a skin disease. Further large-scale studies are needed to find the prevalence of AAA in psoriasis and the appropriate screening time of that in this disease.

## Conflict of Interest

The authors declare that there are no conflicts of interest.
